# Deep learning enables automated MRI-based estimation of uterine volume also in patients with uterine fibroids undergoing high-intensity focused ultrasound therapy

**DOI:** 10.1186/s13244-022-01342-0

**Published:** 2023-01-05

**Authors:** Maike Theis, Tolga Tonguc, Oleksandr Savchenko, Sebastian Nowak, Wolfgang Block, Florian Recker, Markus Essler, Alexander Mustea, Ulrike Attenberger, Milka Marinova, Alois M. Sprinkart

**Affiliations:** 1 Department of Diagnostic and Interventional Radiology, University Hospital Bonn, Venusberg-Campus 1, 53127 Bonn, Germany; 2 Department of Radiotherapy and Radiation Oncology, University Hospital Bonn, Venusberg-Campus 1, 53127 Bonn, Germany; 3 Department of Neuroradiology, University Hospital Bonn, Venusberg-Campus 1, 53127 Bonn, Germany; 4 Department of Gynaecology and Gynaecological Oncology, University Hospital Bonn, Bonn, Germany; 5 Department of Nuclear Medicine, University Hospital Bonn, Bonn, Germany

**Keywords:** Deep learning, Magnetic resonance imaging, Uterus, Leiomyoma

## Abstract

**Background:**

High-intensity focused ultrasound (HIFU) is used for the treatment of symptomatic leiomyomas. We aim to automate uterine volumetry for tracking changes after therapy with a 3D deep learning approach.

**Methods:**

A 3D nnU-Net model in the default setting and in a modified version including convolutional block attention modules (CBAMs) was developed on 3D T2-weighted MRI scans. Uterine segmentation was performed in 44 patients with routine pelvic MRI (standard group) and 56 patients with uterine fibroids undergoing ultrasound-guided HIFU therapy (HIFU group). Here, preHIFU scans (*n* = 56), postHIFU imaging maximum one day after HIFU (*n* = 54), and the last available follow-up examination (*n* = 53, days after HIFU: 420 ± 377) were included. The training was performed on 80% of the data with fivefold cross-validation. The remaining data were used as a hold-out test set. Ground truth was generated by a board-certified radiologist and a radiology resident. For the assessment of inter-reader agreement, all preHIFU examinations were segmented independently by both.

**Results:**

High segmentation performance was already observed for the default 3D nnU-Net (mean Dice score = 0.95 ± 0.05) on the validation sets. Since the CBAM nnU-Net showed no significant benefit, the less complex default model was applied to the hold-out test set, which resulted in accurate uterus segmentation (Dice scores: standard group 0.92 ± 0.07; HIFU group 0.96 ± 0.02), which was comparable to the agreement between the two readers.

**Conclusions:**

This study presents a method for automatic uterus segmentation which allows a fast and consistent assessment of uterine volume. Therefore, this method could be used in the clinical setting for objective assessment of therapeutic response to HIFU therapy.

**Supplementary information:**

The online version contains supplementary material available at 10.1186/s13244-022-01342-0.

## Key points

- Deep learning methods enable accurate segmentation of the uterus in T2-weighted MRI.
- Automatic uterine volumetry is possible in patients with and without leiomyomas.
- Automated volumetry enables an objective assessment of response to high-intensity focused ultrasound therapy.

**Supplementary information:**

The online version contains supplementary material available at 10.1186/s13244-022-01342-0.

## Background

Uterine fibroids, also known as leiomyomas, are the most common benign pelvic tumors in women of reproductive age. Fibroid-associated symptoms are observed in about one-third of affected patients [1]. Major symptoms are severe and extended menstrual bleeding (hypermenorrhea and dysmenorrhea) that may lead to anemia-associated complications. Depending on size and location, uterine fibroids can also cause pelvic pressure, urinary frequency and even incontinence and can be associated with adverse reproductive outcome. Thus, symptomatic uterine fibroids have a negative impact on daily living and quality of life [2, 3].

Current treatment strategies mainly involve surgical interventions as laparoscopic or hysteroscopic myomectomy and laparoscopic hysterectomy [4–6]. Nowadays, organ-preserving minimally invasive and noninvasive therapies are becoming increasingly important. In recent years, high-intensity focused ultrasound (HIFU), guided by either ultrasound or magnetic resonance tomography, has also emerged as a viable effective and low-risk treatment option for symptomatic uterine fibroids [7–11]. During the HIFU procedure, the uterine fibroids are thermally ablated by concentrating the ultrasound energy inside the fibroid leading to thermal coagulation necrosis and additional cavitation damage [9, 12–16]. Previous studies have shown that HIFU treatment of symptomatic leiomyomas results in a significant reduction in uterine fibroid volume and total uterine volume during follow-up. In addition, a correlation between improvement in fibroid-associated symptoms and reduction in uterine fibroid volume has been demonstrated [7, 10, 11]. Therefore, automation of uterine measurements is highly desirable in order to be able to assess the response to treatment objectively, quickly and reproducibly.

In recent years, the utility of machine learning and, in particular, deep learning methods has been demonstrated for various medical tasks including medical imaging. To date, most deep learning approaches in medical imaging use artificial neural networks trained in a supervised manner, which means that model development requires annotated data with the desired outcome, also known as ground truth. One potential application of such deep learning models is the automation of quantitative image analysis, which would otherwise require tedious manual effort. In addition, deep learning methods have also shown great potential for detecting and characterizing pathological findings, which could assist radiologists in making the diagnosis [17–21].

Various deep learning methods have also been proposed for volumetry based on medical image segmentation. A successful neural network for various organ and tissue type segmentation is the open-source framework nnU-Net, a self-adapting pipeline based on the U-Net model introduced by Ronneberger et al. [22–24]. To improve the weighting of the feature map signals, convolutional block attention modules (CBAM) have been suggested, which have led to high performance for various classification, object detection, and segmentation tasks, also in combination with a U-Net architecture [25–28].

Very recently, various neural networks have been proposed for uterine segmentation in MRI and ultrasound imaging, where most of them are also based on the U-Net architecture [29–32]. However, none of these has presented a suitable method for accurate automatic volumetry of the uterus, especially when the evaluation of longitudinal data is required to assess treatment response, such as in patients with uterine fibroids undergoing HIFU therapy.

Against this backdrop, the aim of this study was to develop a 3D deep learning method that allows accurate uterine segmentation of patients with and without uterine fibroids and to ensure automatic assessment of changes in uterine volume during HIFU therapy. For this purpose, two neural networks were trained and compared, a standard 3D U-Net and a modified U-Net using additional CBAMs in the encoder, implemented in the nnU-Net framework.

## Methods

### Dataset

This study was approved by the local Ethics Committees at the Medical Faculty of the Rheinische Friedrich-Wilhelms-Universität Bonn (no. 295/19). Data of 44 consecutive patients without uterine fibroids who received routine pelvic MRI (standard group, mean age 38 ± 13 years) and of 56 patients with uterine fibroids who underwent ultrasound-guided HIFU therapy (HIFU group, mean age 43 ± 6 years) were included. The only inclusion criteria were the availability of an axial 3D T2-weighted MRI acquired with a turbo spin echo sequence at a 1.5 Tesla scanner (Philips Ingenia) with a slice thickness <  = 5 mm and an in-plane voxel size <  = 1 mm. In the HIFU group three examinations per patient were considered: An examination prior to HIFU intervention (preHIFU, *n* = 56), the immediate follow-up examination maximum one day after HIFU therapy (postHIFU_1, *n* = 54), and the last available follow-up examination (postHIFU_Last, *n* = 53). In two cases, there was no early follow-up within one day after HIFU, and in three cases, patients received only one follow-up examination. The mean time interval between HIFU and the last available follow-up examination was 420 ± 377 days (range: [97; 2007]). Overall, a total of 207 scans from 100 patients were used for method development. Additional information on the dataset and the scanning parameters can be found in Table 1.

**Table 1:** Scan and image parameters of the dataset

	Mean	Median	Range
Pixel spacing (mm)	0.39	0.37	[0.33; 0.61]
Spacing between slices (mm)	4.34	4.4	[3.3; 4.95]
Slice thickness (mm)	3.95	4	[3; 4.5]
Matrix size	1004	1024	[704; 1280]
Number of slices	42	40	[40; 60]
Echo time (TE) (ms)	90	90	[90; 90]
Repetition time (TR) (ms)	3922.15	3729.70	[3729.70; 5594.55]

The ground truth for the preHIFU images was generated by a board-certified radiologist (O.S., 9 years of experience in radiology and 4 years of experience in gynecologic imaging) and additionally by a radiology resident (T.T., in his fourth year of residency with 2 years of experience in gynecologic imaging). Contours of the uterus were outlined in all slices using the open-source software 3D Slicer [33]. To assist the generation of the ground truth for the remaining datasets, a default 3D nnU-Net was trained on the segmentations of the preHIFU dataset from the board-certified radiologist [23]. This early model was applied to all follow-up examinations of the HIFU group and to the standard non-fibroid group, and the predicted segmentations were subsequently adapted manually by the radiology resident or the board-certified radiologist. The board-certified radiologist approved all segmentations of the resident.

For method development, datasets of both the standard and the HIFU group were randomly divided into 80% training and 20% test cases, where a single patient was included completely either in the training or in the test set, resulting in 169 training and 38 hold-out test cases (see Table 2).

**Table 2:** Number of datasets in the training and test sets for the different groups

	Standard group	HIFU group		
		preHIFU	postHIFU_1	postHIFU_Last
Training	36	45	45	43
Test	8	11	9	10

### Model

**Fig. 1:**
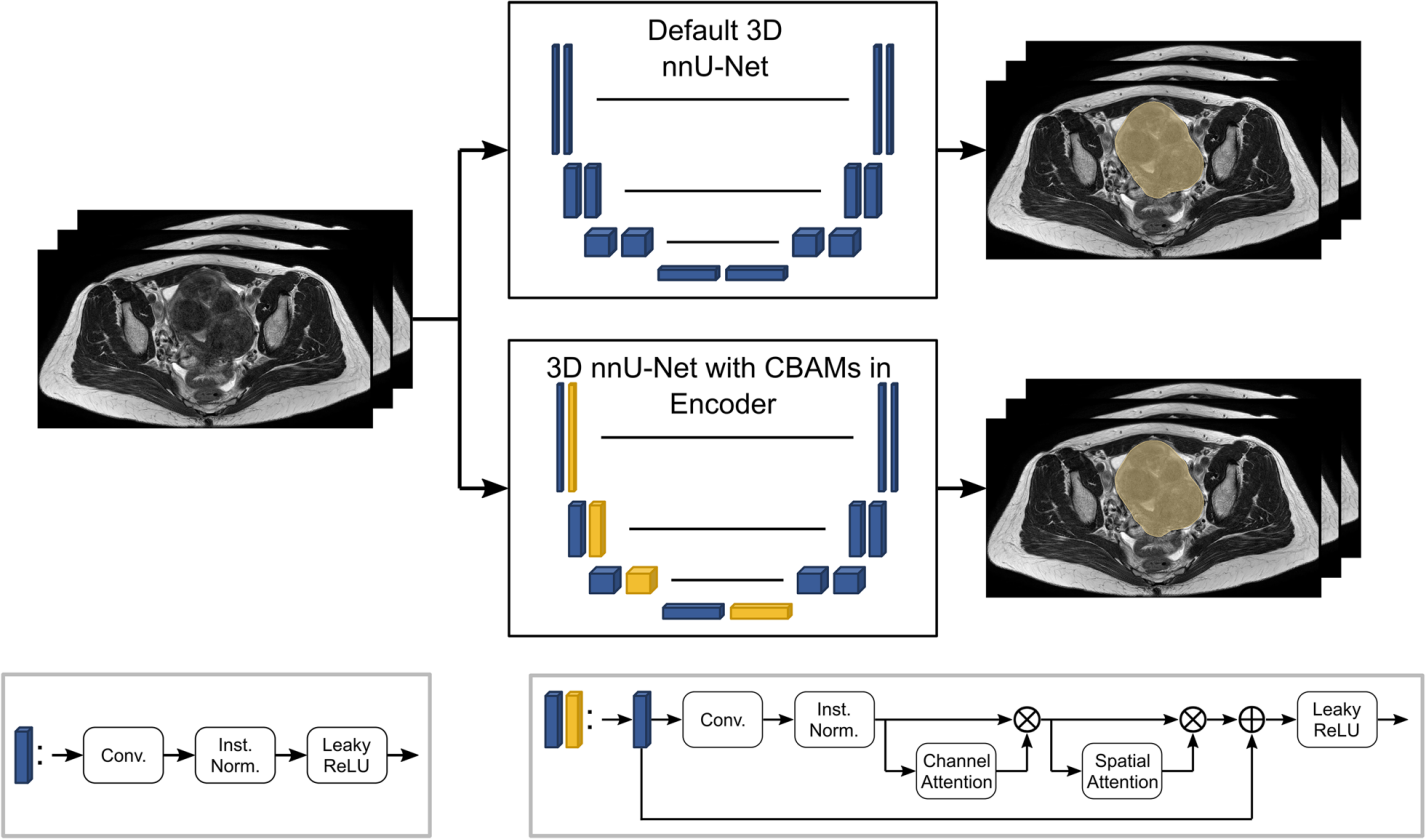
Schematic illustration of the used architectures: One layer in the default nnU-Net configuration consists of a convolution followed by an instance normalization and a leaky ReLU activation (blue). The modified architecture investigated in this work contains layers with additional CBAMs in the encoder (yellow), consisting of a convolution and an instance normalization followed by a channel and a spatial attention module, each connected by element-wise multiplication. The output of the first convolutional block and the output of the CBAM are merged by element-wise summation and again activated by leaky ReLU activation function

For automatic uterine segmentation, two different deep learning architectures were trained for 500 epochs based on the 3D nnU-Net framework, one in the default setting and the other with additional CBAMs in the encoder [23, 25]. The default nnU-Net architecture is generated based on the fingerprint of the training dataset, which determines several preferences and parameters, e.g., preprocessing, network depth and the kernel size of the convolutional layers. However, the general architecture of the encoder and decoder always consists of two blocks per resolution step, where one block consists of a convolution, an instance normalization and a leaky rectified linear unit (ReLU) activation [23]. This default architecture was compared to a modified version, where the second block in the encoder was replaced with a CBAM [25]. A CBAM layer returns a weighted feature map, in which important signals should be enhanced and unimportant ones suppressed. In principle, this should improve the focus on the relevant image information and its location [15]. The use of CBAM in combination with a nnU-Net layer is illustrated in Fig. 1.

Using CBAMs in the nnU-Net encoder increases the number of trainable parameters by only 0.12%. This still allows for a relatively fair comparison of both architectures. For more details on the two investigated architectures, see Additional file 1: S1.

### Evaluation

Both models were trained with fivefold cross-validation; thus, the entire training dataset was split into five validation sets and a single model was trained on each of the remaining training data, resulting in five different models for each of the two investigated methods. The performance of the two architectures was determined based on the mean performance of the five validation sets.

Agreement to the ground truth was measured in terms of Dice score and relative volume difference. The final model was evaluated on the hold-out test data ensembling the predictions of the five individual models from cross-validation. Inter-reader agreement was determined for the preHIFU scans. Model performance was compared to the human inter-reader agreement based on the hold-out test samples from that group.

To investigate whether the model is suitable for post-HIFU treatment follow-up, the automatically determined volume difference between before and after HIFU was compared with the ground truth. All follow-up scans from the HIFU group included in the hold-out test data were considered in this analysis. Pearson correlation coefficient was determined and a Bland–Altman analysis was performed using the Python packages seaborn and pyCompare [34, 35]

## Results

**Fig. 2:**
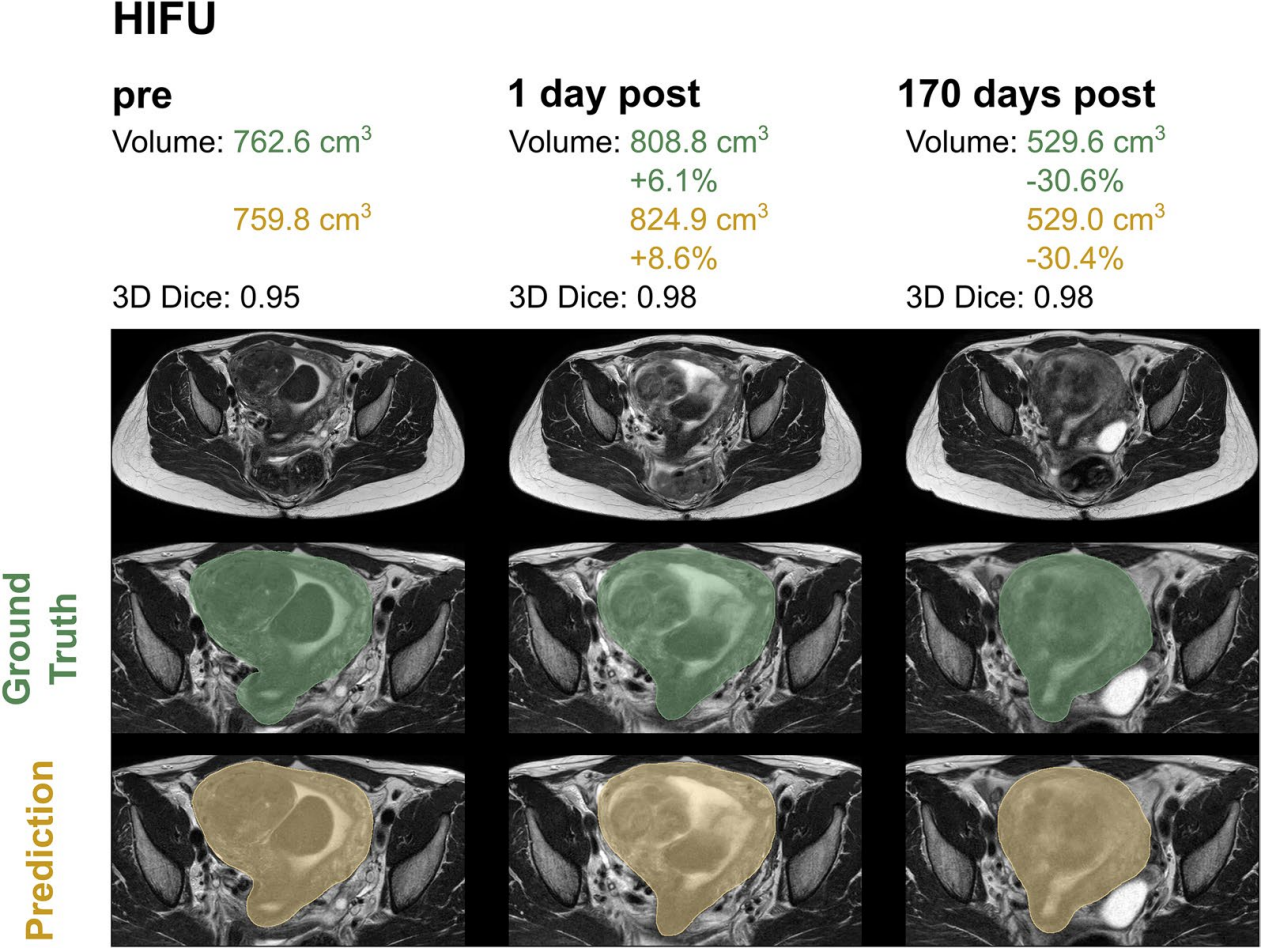
Uterine segmentation in a patient undergoing HIFU therapy: before treatment, one day after treatment and 170 days after treatment. Automatic segmentation achieved with the default 3D nnU-Net is shown in yellow and ground truth segmentation validated by a board-certified radiologist in green

**Fig. 3:**
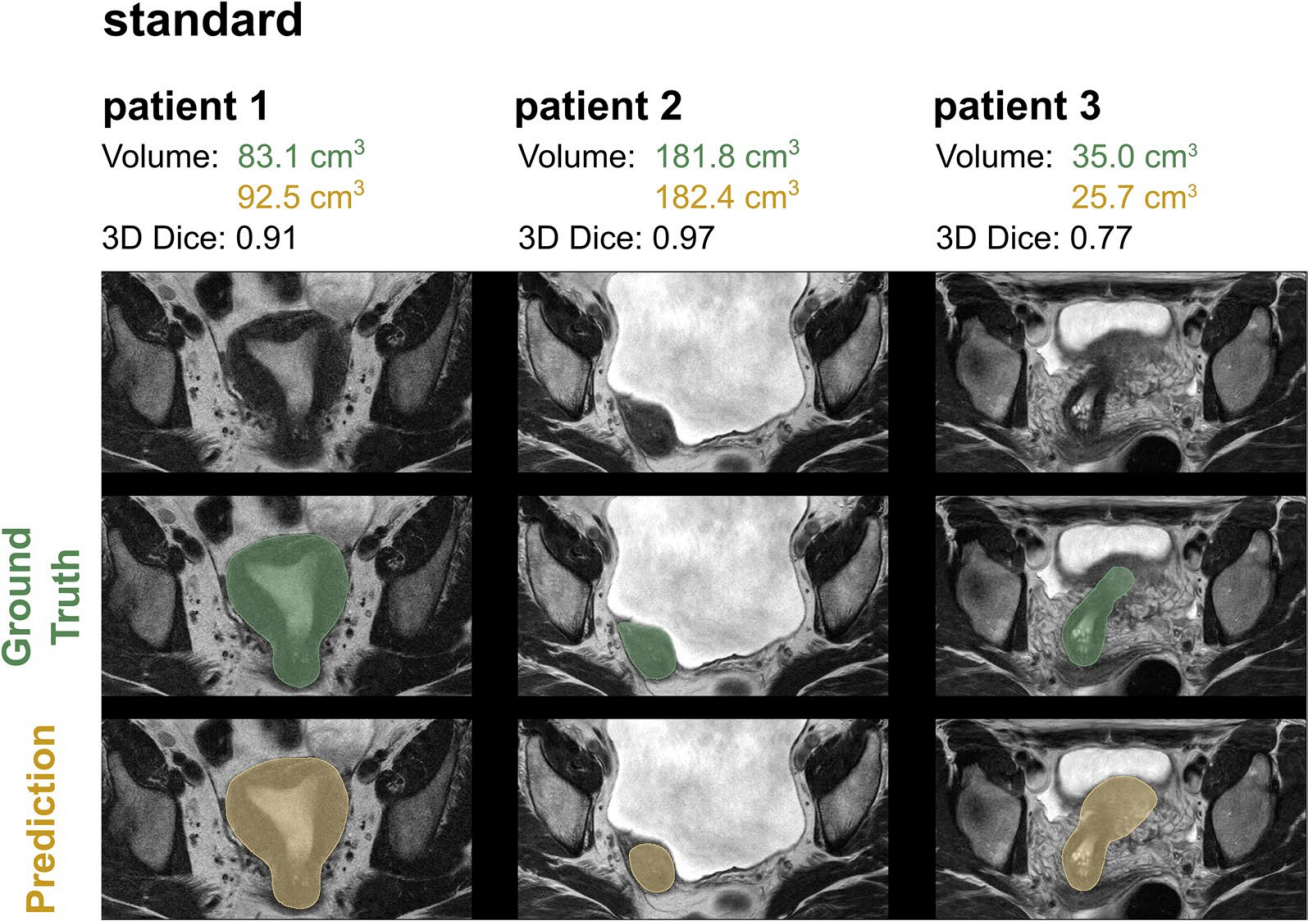
Predicted uterine segmentation of the default 3D nnU-Net (yellow) and the ground truth segmentation verified by the board-certified radiologist (green) of three different patients in the standard group without uterine fibroids

For both model architectures investigated, an excellent uterine segmentation performance was observed in the standard and all HIFU groups. Figure 2 shows an example for predicted and ground truth uterine segmentation of a patient with uterine fibroids prior to HIFU, short after and 170 days after HIFU treatment. Figure 3 shows three patients of the standard group without uterine fibroids.

The mean performance on each of the five validation sets was very similar for the two models, with a mean relative volume difference of 3.79% for the default nnU-Net and 3.70% for the CBAM nnU-Net and a mean Dice score of 0.95 for both architectures. Since the use of CBAM did not lead to a significant benefit compared to the default nnU-Net configuration for the task of uterus segmentation, the less complex default architecture was chosen as the final segmentation model which was applied to the hold-out test data. A detailed comparison of the two architectures can be found in Additional file 1: S2.

The ensemble of the fivefold cross-validated 3D nnU-Net with default settings applied to the hold-out test data resulted in a mean Dice score of 0.95 and a mean relative volume difference of 4.08% (Table 3).

**Table 3:** Mean Dice scores and mean relative volume difference reported for the entire hold-out test data and separately for the preHIFU dataset, the early (postHIFU_1) and last follow-up (postHIFU_Last) after HIFU treatment, as well as for the non-fibroid standard group

Dataset	Dice score	Relative volume difference
All (*n* = 38)	0.95 ± 0.04	4.08% ± 4.86%
preHIFU (*n* = 11)	0.94 ± 0.02	3.63% ± 2.91%
postHIFU_1 (*n* = 9)	0.97 ± 0.01	2.15% ± 1.50%
postHIFU_Last (*n* = 10)	0.97 ± 0.02	3.12% ± 2.93%
Standard group (*n* = 8)	0.92 ± 0.07	8.09% ± 8.61%

For the preHIFU dataset, annotated data of both readers were available (*n* = 56). The inter-reader comparison between the board-certified radiologist and the radiology resident on this dataset showed a mean Dice score of 0.92 and a mean relative volume difference of 4.03%. The comparison of the inter-reader agreement and the agreement of both readers with the final segmentation model on the preHIFU test data is listed in Table 4. Comparing the predictions of the neural network with the segmentations of the two readers shows a mean Dice score of 0.91 and higher, indicating a segmentation performance of the neural network similar to human performance.

**Table 4:** Comparison of inter-reader agreement between the board-certified radiologist (reader 1) and the intensively trained radiology resident (reader 2) and the respective agreement of these readers with the predicted segmentations of the nnU-Net on the preHIFU data in the hold-out test set (*n* = 11)

	Dice score	Relative volume difference
Reader 1 vs. Reader 2	0.92 ± 0.02	3.69% ± 3.54%
Reader 1 vs. nnU-Net	0.94 ± 0.02	3.63% ± 2.91%
Reader 2 vs. nnU-Net	0.91 ± 0.03	4.49% ± 5.26%

The table provides the mean Dice scores and the mean relative volume difference

**Fig. 4:**
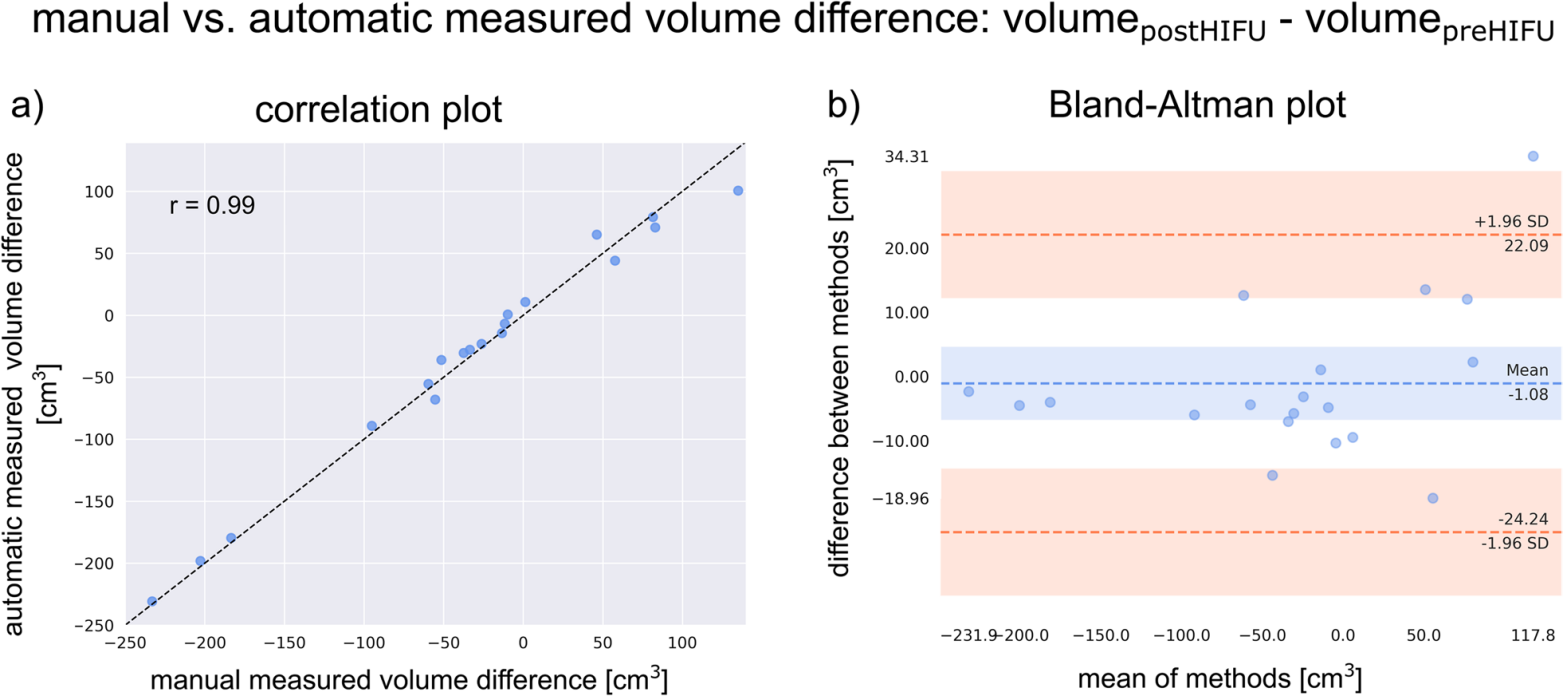
Scatterplot with Pearson correlation coefficient (**a**) and Bland–Altman plot (**b**) for agreement analyses between automatic measured volume difference and ground truth before and after HIFU therapy

The agreement between ground truth and automatically determined volume difference before and after HIFU treatment was compared using all follow-up data of the HIFU group included in the hold-out test (*n* = 19). A strong correlation was observed with a Pearson correlation coefficient of 0.99. The Bland–Altman analysis shows a mean difference of -1.08 cm^3^ (see Fig. 4).

## Discussion

This work presents an innovative method for deep learning-based 3D segmentation of the uterus for automatic and accurate determination of uterine volume in T2-weighted MRI scans with the focus on being applicable in uterine fibroid patients undergoing HIFU treatment. To achieve this, a dataset was used for method development and evaluation that included a relevant portion of examinations prior to HIFU therapy and at different timepoints thereafter. In order to enrich the dataset and to investigate the applicability of uterus segmentation to routine MRI scans, standard examinations from clinical routine were also included. The network performed very well for both extremes, i.e., patients without fibroids and patients with multiple symptomatic fibroids of different sizes who were candidates for HIFU ablation. Thus, it may be assumed (although not proven in this study) that high-quality uterine segmentation can be achieved also in patients with smaller, non-symptomatic fibroids. The performance in the HIFU group was slightly higher than that in the standard group. This may be explained in part by the lower absolute uterine volume in patients without uterine fibroids, which results in lower Dice scores in areas where partial volume effects make exact delineation of uterine tissue difficult.

In routine clinical practice, the volume of uterus and uterine fibroids has been assessed by magnetic resonance imaging and is currently measured either from the diameters in the anterior–posterior, cranio-caudal and right–left directions using the volume formula of a prolate ellipsoid [7] or by manually drawing the contours on each axial slice [10]. Therefore, an accurate volumetry is very time-consuming, so that automation of the measurement is very desirable.

Two different approaches for automatic uterine segmentation were compared in this study: A default 3D nnU-Net and a modified version with CBAMs in the encoder. The advantage of CBAMs has already been shown in various works for classification, object detection and segmentation tasks [25–28]. Although the CBAM nnU-Net architecture has shown excellent performance on the five validation sets, it could not outperform the default architecture overall, which may be attributed to its already high segmentation performance. It may nevertheless be worthwhile to further investigate the combination of CBAM and U-Net models for different medical segmentation tasks, especially in cases where the performance of a standard U-Net is limited.

In order to compare the performance of the nnU-Net model with that of human readers, manual segmentations were generated independently by both annotators and compared to the automatic segmentation on a subset of the data. This comparison showed a similar level of agreement between the board-certified radiologist, the intensively trained resident and the automatic measurements.

Previous research has already investigated deep learning methods for uterine segmentation, where most of the presented approaches are also based on a U-Net architecture [29–31]. In one of these studies, also based on MRI, a 3D U-Net model requiring only minimal user interaction for the segmentation of the uterine cavity and the placenta of pregnant women was presented and evaluated in normal pregnant women and also in women with suspected placental abnormalities. Mean Dice scores of 92% and 88% were achieved for uterine cavity segmentation in the two groups [29].

For ultrasound images, automated segmentation approaches have also been proposed using a modified 2D U-Net architecture for segmentation of the uterus [30]. Patients with uterine fibroids were not specifically considered in that study and although several models were trained at different 2D planes, overall only low Dice scores were reported. The authors attribute this to issues with slices near the uterine edge, demonstrating the limitation of 2D approaches. To a certain extent, the lower segmentation performance may also be explained by the quality of the available image data, which essentially depends on the sonographic experience of the examiner.

A further approach, also based on MRI, uses a DenseUNet for segmentation of the uterus on sagittal slices [31]. That work proposed the sharpening of uterine edges in a preprocessing step, which was added as additional input to the network, leading to a mean Dice score of 87.6%. This 2D approach was also not developed in patients with uterine fibroids.

In contrast, Zhang et al. have presented the HIFUNet, an encoder–decoder network with a pre-trained ResNet101 [36] encoder for segmentation of the uterus, uterine fibroids and the spine on 2D sagittal MRI slices. This study included only preoperative patients and was employed for HIFU therapy planning. Precise segmentation was reported to be difficult at the margins for patients with many fibroids, resulting in a Dice score for uterine segmentation of 82.37%. The authors suggest that direct 3D segmentation may lead to higher segmentation performance [32]. Because post-HIFU image data were not included in this study, information on the applicability of this method for accurate assessment of treatment-related volume changes over time after HIFU ablation is missing. However, a clear advantage of the HIFUNet is the simultaneous segmentation of individual uterine fibroids, which has not been addressed in the current study so far. Direct region detection of uterine fibroids after MRI-fused ultrasound using a combination of split-and-merge and multi-seed region growing methods was also considered in another work [37]. These studies allow direct segmentation of single fibroids, thus enabling immediate assessment of the treatment response of individual fibroids.

It should be noted that the precise contours, especially of small fibroids, are often difficult to delineate from adjacent uterine tissue, even for human readers. This may also contribute to lower accuracy of segmentation of individual fibroids compared to uterine segmentation [32, 37]. From a clinical perspective, segmentation of the entire uterus may already be a relevant measure for therapy response assessment. For example, when multiple fibroids are treated, the improvement in myoma-associated symptoms is probably primarily related to the reduction in overall uterine volume. Thus, in the post-interventional course, the uterus may lie differently in the pelvic region due to the reduction in its total size, i.e., no longer pressing on the urinary bladder or the Fallopian tubes. The segmentation of the entire uterus provided by our approach is a fast, accurate, and reproducible method that can be applied on MRI image data also in the post-interventional course, even without knowledge of the number and exact location of the treated fibroids. In many cases, the latter is only known to the therapist. Nevertheless, the presented method may also serve as an input for targeted segmentation of single fibroids for assessment of treatment response.

Our study has several limitations. First, part of the labeled dataset was generated semi-manually, in which a network trained on a subset of the data was used for deep learning-assisted annotation. However, all data used as ground truth were finally validated by a board-certified radiologist. In addition, the deep learning model was specifically trained for axial T2-weighted turbo spin echo sequences with explicit specifications regarding the spatial resolution. The study was performed in only one center from a single MRI scanner. Therefore, the generalizability should be further evaluated in a multicenter setting. The use of the algorithm will be enabled for collaborative studies on reasonable request.

## Conclusion

This study provides a method for automatic segmentation of the uterus from patients with and without uterine fibroids with a performance similar to human readers, enabling fast, easy and reproducible assessment of volume changes in the clinical setting of a HIFU therapy.

## Supplementary information

**Additional file 1. S1**. Details on method development.** S2**. Comparison of both architectures.

## Data Availability

The datasets analyzed during the current study are not publicly available due to data protection laws.

## Abbreviations

CBAM: Convolutional block attention module
HIFU: High-intensity focused ultrasound
ReLU: Rectified linear unit
