# Supplementary material for: Deep learning enables automated MRI-based estimation of uterine volume also in patients with uterine fibroids undergoing high-intensity focused ultrasound therapy
Source: Insights Imaging. 2023 Jan 5;14:1. doi: 10.1186/s13244-022-01342-0 (PMC9813298; doi:10.1186/s13244-022-01342-0)
Supplement: Supplementary file 1 — Additional file 1. S1. Details on method development. S2. Comparison of both architectures. [file 13244_2022_1342_MOESM1_ESM.pdf]

ELECTRONIC SUPPLEMENTARY MATERIAL

Deep learning enables automated MRI-based estimation of uterine volume also in patients with uterine fibroids undergoing high-intensity focused ultrasound therapy

S1 Details on method development

The implementation of the nnU-Net framework was taken from https://github.com/MIC-DKFZ/nnUNet and the “3D fullres” configuration was selected for training the models. For the default nnU-Net, training was performed with mixed precision on an Nvidia GeForce RTX 3060 Grahpics Processing Unit (GPU) with 12 gigabyte video memory. Except for the number of epochs, which was reduced to 500, no further changes were made to the default settings for training the 3D nnU-Net. Thus, training was performed with a stochastic gradient descent optimizer, with a nesterov momentum of μ = 0.99 and an initial learning rate of 0.01, which was decreased after each epoch by following the poly learning rate scheme. For optimization a combined loss function of cross-entropy and Dice loss was used. All pre-processing steps, including normalization and resampling to the median resolution (0.372 x 0.372 x 4.4 mm), as well as data augmentation (e.g. rotation, scaling, Gaussian noise and blur, …) were handled by the nnU-Net framework [1]. The exact network architecture as well as input patch and batch size is automatically specified by the framework based on the used dataset. In each encoder and decoder step, two convolutional blocks were applied, where one block consists of convolution, followed by instance normalization and leaky rectified linear unit (ReLU) activation function. Here, a batch size of 2 and a patch size of 384x320x14 were determined by the nnU-Net. The resulting architecture consists of six encoder and decoder blocks and one bottleneck block. In the first three encoder and last two decoder blocks, a 3x3x1 convolutional kernel was applied for handling anisotropic spacing in z-direction. For all other convolutions, a kernel size of 3 was used for each dimension. Down-sampling was performed with strided convolutions and up-sampling with transposed convolutions. All encoder and decoder blocks were concatenated by skip connections. In addition, deep-supervision was used by applying a 1x1x1 convolutional kernel with softmax activation function at each resolution step, except for the lowest resolution. For each deep supervision output, the loss was calculated and the final loss was obtained from a weighted sum of all these losses [1]. The resulting architecture is also illustrated in Figure S1, where only the second convolutional block in the encoder has to be replaced by the previous one and the stride of the convolution has to be changed to 1 for each dimension.

For the modified version, which was investigated in this study, the second convolutional kernel in each encoder step was replaced with convolutional block attention modules (CBAMs) [2]. The use of CBAMs was also investigated for detection of cerebral aneurysms, where also an implementation of this module was provided (https://github.com/CTA-detection/) [3]. This implementation was used for modifying the nnU-Net architecture in this study. Apart from this change, no further modifications to the default architecture were made. In contrast to the default 3D nnU-Net, the CBAM nnU-Net was trained on an Nvidia GeForce RTX 3090 GPU with 24 gigabyte video memory without mixed precision, as mixed precision resulted in unstable training. One CBAM consists of a channel and a subsequent spatial attention module. The channel attention module, focusing on what is the relevant part in the image, performs a squeezing of the input feature map along the spatial dimension and aggregates features by using both average and max pooling operations. The resulting spatial context features are used as input to a multi-layer perceptron with one hidden layer. The output of this multi-layer perceptron for both features is concatenated by element-wise summation and is activated using ReLU activation function. In contrast to the channel

attention, the spatial attention focuses on where the informative part is located in the image. This information is derived by applying a 7x7 convolutional layer to the concatenation of an average- and max-pooling operation along the channel axis, where the output is again activated using ReLU [2].

Overall, a convolution with subsequent instance normalization is first applied in the modified second convolutional block in the encoder. This output is afterwards processed by the channel attention module, where the resulting feature map is linked to the output of the instance normalization by element-wise multiplication. Next, the spatial attention module is applied to this feature map. Again, the output of the channel and the spatial attention are combined by element-wise multiplication. This weighting of the output features is intended to suppress unimportant signals and amplify important ones. Finally, the resulting feature map is combined with the output of the first convolutional block by element-wise summation and activated by leaky ReLU activation function.

Since no other changes were made to the default architecture, all convolution kernel sizes as well as the strides for down-sampling and the settings of the transposed convolutions for up-sampling correspond to the resulting default 3D nnU-Net architecture (see Figure S1).

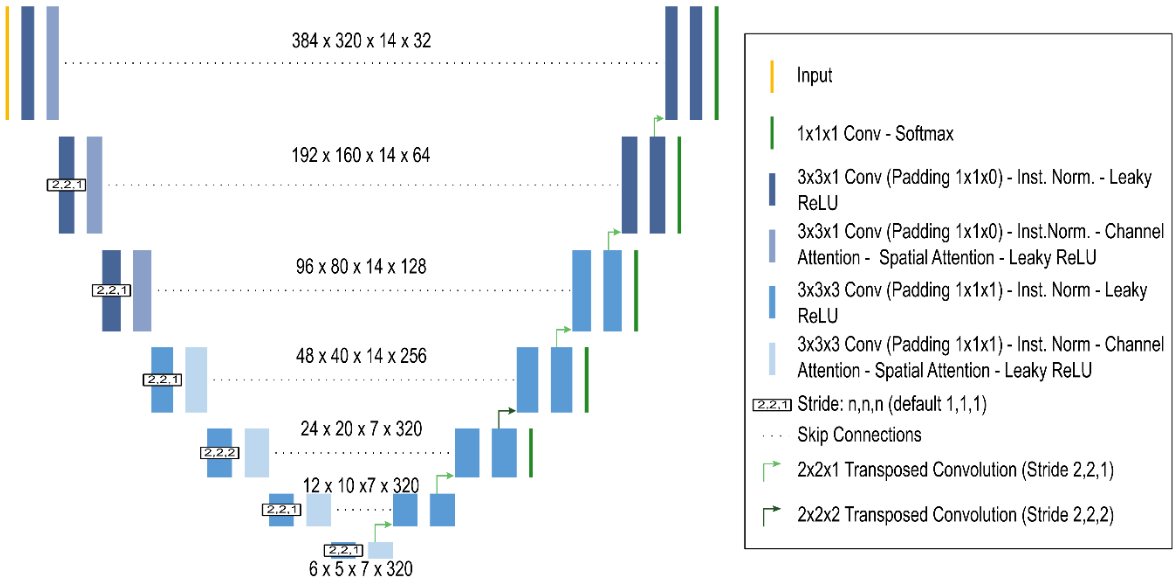

Figure S1: Architecture of the resulting CBAM 3D U-Net using the nnU-Net framework

S2 Comparison of both architectures

The CBAM nnU-Net and the default nnU-Net achieved similar results on the five validation sets (see Table S1).

Dataset	nnU-Net	Dice-score	Relative volume difference

**All (n = 169)**	Default	0.95 ± 0.05	3.79% ± 6.49%
	CBAM	0.95 ± 0.04	3.70% ± 5.84%
**preHIFU (n = 45)**	Default	0.94 ± 0.02	3.49% ± 2.83%
	CBAM	0.94 ± 0.02	3.41% ± 2.91%
**postHIFU_1 (n = 45)**	Default	0.97 ± 0.01	1.89% ± 1.80%
	CBAM	0.97 ± 0.01	1.91% ± 1.77%
**postHIFU_Last (n = 43)**	Default	0.96 ± 0.03	2.32% ± 4.30%
	CBAM	0.96 ± 0.03	2.62% ± 4.83%
**Standard group (n = 36)**	Default	0.91 ± 0.07	8.28% ± 11.74%
	CBAM	0.91 ± 0.06	7.57% ± 10.00%

Table S1: Mean Dice-score and mean relative volume difference on the five validation sets reported separately for each dataset and for the default nnU-Net and the modified nnU-Net with CBAMs in the encoder.

The similar performance of both trained models is also visible in Figure S2. Thus, the modified CBAM nnU-Net investigated in this study could not lead to a better segmentation result compared to the default model.

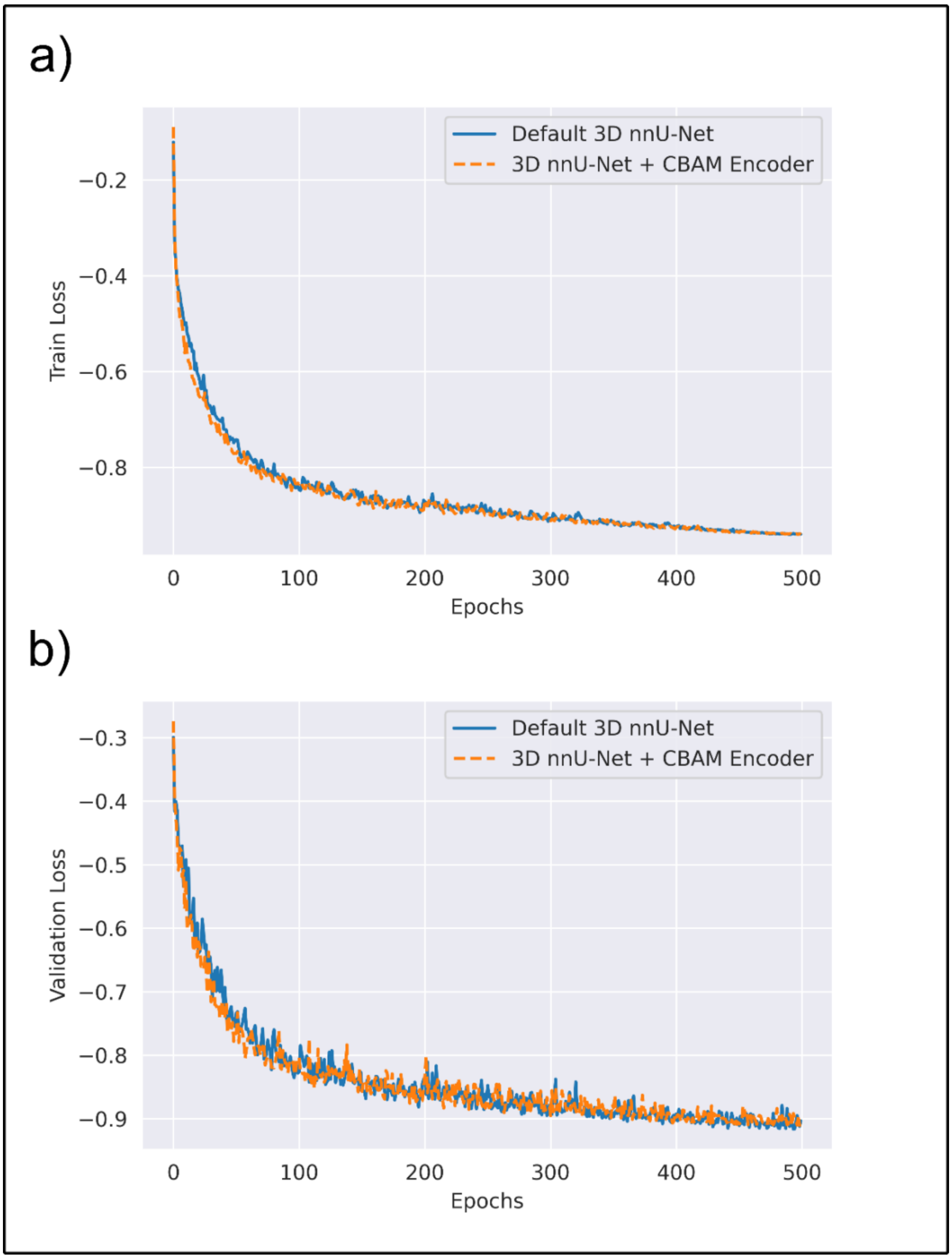

Figure S2: Train (a) and validation loss (b) provided for the default 3D nnU-Net and for the modified CBAM nnU-Net.

References

1. Isensee F, Jaeger PF, Kohl SAA, Petersen J, Maier-Hein KH (2021) nnU-Net: a self-configuring method for deep learning-based biomedical image segmentation. Nat Methods 18:203–211.

2. Woo S, Park J, Lee J-Y, Kweon IS (2018) CBAM: Convolutional Block Attention Module. Proceedings of ECCV 2018:3–19

3. Yang J, Xie M, Hu C, et al (2021) Deep Learning for Detecting Cerebral Aneurysms with CT Angiography. Radiology 298:155–163.
